# Editorial: Internet of Medical Things and computational intelligence in healthcare 4.0

**DOI:** 10.3389/fdata.2024.1368581

**Published:** 2024-02-21

**Authors:** Sujata Dash, Subhendu Kumar Pani, Wellington Pinheiro dos Santos

**Affiliations:** ^1^Department of Information Technology, School of Engineering and Technology, Nagaland University, Dimapur, India; ^2^Department of Computer Science and Engineering, Krupajal Engineering College, Bhubaneswar, India; ^3^Department of Biomedical Engineering, Federal University of Pernambuco, Recife, Brazil

**Keywords:** computational intelligence, Internet of Medical Things (IoMT), healthcare 4.0 applications, biomedical informatics, machine learning, deep learning

We are delighted to present this special editorial, exploring the dynamic Research Topic of “*Internet of Medical Things and computational intelligence in healthcare 4.0*.” As we navigate the intricate realms of healthcare's digital transformation, the synergy between the Internet of Things (IoT) and computational intelligence stands as a beacon of innovation, promising a paradigm shift in the way we perceive, deliver, and experience healthcare. Healthcare 4.0 encapsulates a vision where interconnected devices, advanced analytics, and artificial intelligence converge to create a holistic, patient-centric ecosystem. At the heart of this transformative journey lies the intersection of the Internet of Medical Things (IoMT) and Computational Intelligence, propelling healthcare into an era marked by unprecedented efficiency, personalized care, and empowered patients.

The Internet of Medical Things (IoMT) has seen substantial growth, with an increasing number of connected healthcare devices. According to a report by Grand View Research, the global IoMT market size was valued at over USD 44 billion in 2020 and is expected to exhibit a compound annual growth rate (CAGR) of around 19.2% from 2021 to 2028. This underscores the rapid adoption of IoMT technologies in healthcare systems worldwide. Additionally, a study by Allied Market Research highlighted that the computational intelligence market in healthcare is also on the rise.

There was a large diversity of submissions covering different aspects, from IoMT, Computational Intelligence, Patient-Centric Care in the Digital Age, Ethical Considerations and Security Challenges, and Future Horizons and Collaborative Innovation. The articles and insights shared herein reflect the dedication and innovation of researchers who are at the forefront of this transformative journey. Moreno et al. employ a pattern-based classification method on the African-American Study of Chronic Kidney Disease with Hypertension dataset, revealing 15 distinct clinical features and SNP patterns. Notably, four clinical features and two SNPs show high predictive accuracy for CKD progression. These findings promise to inform future research and advance therapeutic interventions for individuals with chronic kidney disease.

Sharafutdinov et al. propose a new method to assess the generalization of ML models across hospitals. The study shows how the method works using patient data from different hospitals. The results highlight the importance of evaluating model transferability and creating diverse datasets.

Liu et al. analyzed SEER database data from 2004 to 2015 to explore prognostic factors in pancreatic cancer metastasis to the liver across different age groups. They found gender-specific primary sites and age-dependent prognostic factors such as tumor grade, histology, treatment, AJCC N stage, and race. The study underscores the importance of age-specific treatment strategies for pancreatic cancer metastasis to the liver.

Toubiana et al. published an editorial on the use of blockchain for Electronic Vaccine Certificates (EVCs) for COVID-19 vaccination. Blockchain may not be the best solution for EVCs, and the authors suggest exploring alternative cryptographic methods that involve centralized authorities for practical use.

Merhbene et al. used NLP on Reddit data to detect burnout. Their ensemble classifier achieved 0.93 balanced accuracy, outperforming single classifiers. NLP is a highly effective tool for identifying burnout indicators and improves standard classifiers.

Gao et al. reviewed the oral microbiome's relationship with systemic autoimmune diseases (SADs) like SLE, RA, and SS. The review highlights the importance of multiomics data and emphasizes the need for standardized methodologies to improve the understanding of SADs' etiology and potential therapies.

Rath et al. used imbalanced ECG samples to train ML models for detecting HD. AdaBoost and LR outperformed other classifiers. The ensemble model achieved the best HD detection performance. The methodology is versatile and applicable to various disease detection scenarios.

Mishra et al. developed a novel technique for detecting COVID-19 using phoneme analysis and audio signal smearing. They proposed a classification system based on phoneme grouping and achieved 97.22% accuracy for specific phoneme grouping using machine learning classifiers. This technique shows promise for quick and effective early-stage disease detection, with potential for application in other speech-related diseases.

Chicco and Jurman stress the importance of validating supervised machine learning results in biomedical informatics. The challenge is to achieve reliable results in the face of over-optimistic findings. Past guidelines have been too complex, especially for beginners. In response, Walsh et al. (2021) proposed ABC tips to simplify validation. The tips are meant to provide an effective tool for practitioners of all levels to enhance the reliability of scientific results in biomedical sciences.

The market for IoT healthcare is projected to grow at a CAGR of 21.2% from 2024 to 2030, with a value of USD 44.21 billion expected in 2023. This growth is driven by several factors, including the use of wearables and smartphones for patient monitoring, an increased adoption of remote patient monitoring during the COVID-19 pandemic, and investments in digital health infrastructure. The prevalence of chronic conditions and investments in digital healthcare technologies have also led to a significant rise in telemedicine adoption. IoT technology is expanding in healthcare due to several reasons, including advancements in smartphone technology, improved data security measures, and the growing accessibility of wearable sensors and connected health monitors. This demand is further fueled by emerging economies such as India, China, Indonesia, Bangladesh, and some African and Latin American countries, which are contributing to this growth through improvements in network infrastructure and growing network coverage. Physicians are also increasingly using mobile devices, creating further demand for IoT solutions in healthcare.

## Author contributions

SD: Writing – original draft, Writing – review & editing. SP: Writing – review & editing. WS: Writing – review & editing.
